# Rare Giant Angiokeratoma of the Vulva: A Case Report

**DOI:** 10.4274/balkanmedj.2014.0724

**Published:** 2017-03-28

**Authors:** Fatih Doğan, İbrahim Hakan Bucak

**Affiliations:** 1 Department of Plastic Surgery, Adıyaman University School of Medicine, Adıyaman, Turkey; 2 Department of Pediatrics, Adıyaman University School of Medicine, Adıyaman, Turkey

**Keywords:** Angiokeratoma fordyce, vulva, childhood

## Abstract

**Background:**

Angiokeratoma of fordyce occurring over on the vulva is a rare condition. Fordyce angiokeratoma is observed more frequently among men than women. In women, it is generally observed in later life, and appears as multiple dark purple papules, measuring 2-4 mm, on the vulva.

**Case Report:**

We present the case of a 17-year-old white teenage girl with giant Fordyce angiokeratoma on the right vulva. The angiokeratoma was removed and a V-Y advancement flap was made.

**Conclusion:**

In the literature, this is the first childhood case reported in which a reconstruction of the vulva was performed.

Angiokeratomas are benign tumours characterized by ectasia of blood vessels in the papillary dermis associated with acanthosis and hyperkeratosis of the epidermis There are four widely recognized types; solitary angiokeratoma (oral cavities and lower limbs), Fordyce angiokeratoma (on the scrotum or vulva), Mibelli angiokeratoma (on the dorsal skin of fingers and the interdigital area) and angiokeratoma corporis diffusum (on the lower abdomen, genitals, hips and thighs) (1). Fordyce angiokeratoma is observed more frequently among men. In women, it is generally observed in the later stages of life, and appears as multiple dark purple papules, measuring 2-4 mm, on the vulva. In this report, we describe a 17-year-old female patient with a giant Fordyce angiokeratoma, and its surgical reconstruction.

## CASE PRESENTATION

**Figure 1:**
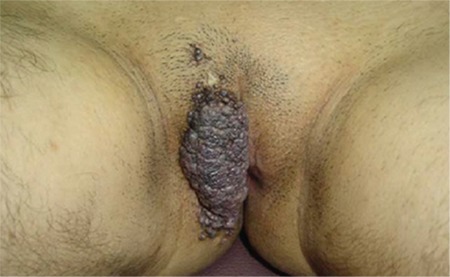
Giant solitary lesion on the right vulva.

**Figure 2:**
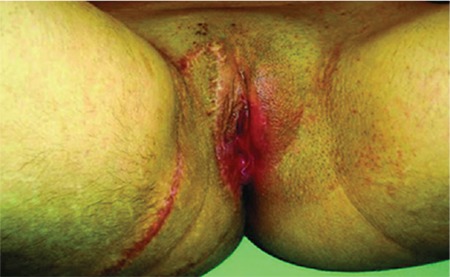
Reconstruction region viewed six months after surgery. The scar is aesthetically acceptable and the vagina inner wall is minimally exposed.

**Figure 3:**
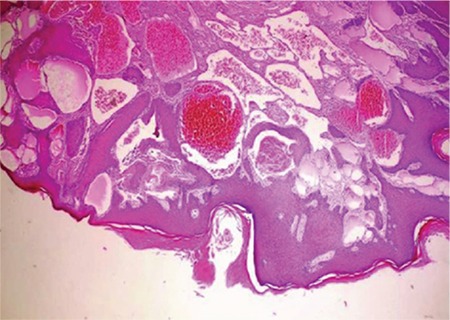
Mild hyperkeratosis, acanthosis in the epidermis with elongation of the rete ridges, and dilated vascular structures in the papillary dermis.

A 17-year-old female patient was admitted to our clinic due to a vulvar, itchy, occasionally bleeding tubercle. Which had been present since her childhood. On her right vulva, there was a purple, solitary lesion with the dimensions of 15x7x4 cm that completely covered the labia major by extending through the perianal region (Figure 1). An incisional biopsy was carried out and histopathologic diagnosis was Fordyce angiokeratoma. The patient's written consent was taken to carry out a surgical procedure. There were no predisposing factors such as pregnancy, postpartum period, radiation to genital region or vulvar varicocele. Under general anesthaesia the lesion was excised together with the right vulva. Internal pudendal artery perforators were detected using a Doppler ultrasound in the right inferior gluteal fold area. Incision of the V-Y advancement flap was made through the subfascial plane in a way that it covered the internal pudendal artery perforators. Reconstruction was satisfactory, with no major complications. The donor-site scar was concealed on the gluteal fold and was aesthetically acceptable (Figure 2). Mild hyperkeratosis, acanthosis in the epidermis with elongation of the rete ridges, and dilated vascular structures in the papillary dermis were observed in the histopathologic examination of the lesion (Figure 3). The written informed consent was taken from the patient’s parents.

## DISCUSSION

Fordyce angiokeratomas are asymptomatic, benign tumours of the papillary dermis and were first described by J.A Fordycea in 1896 (2). These lesions are rare among women and observed as multiple, 2-4 mm papillary structures of similar sizes. The disease may be related to an increase in venous pressure in situations such as contraceptive pill usage and pregnancy (3,4). While in Mibelli disease the autosomal dominant inheritance is related to metabolic diseases, such as angiokeratoma corporis diffusum, also called Fabry disease, no such relation exists in Fordyce angiokeratoma. As Fordyce angiokeratoma could be confused with benign and malignant lesions, such as basal cell carcinoma, vulvar intraepithelial neoplasia, pyogenic granuloma, condyloma acuminata and seborrheic keratosis, histopathological evaluation is the main criterion for diagnosis (5). Generally, Fordyce angiokeratoma does not require medical treatment. Simple excision, cryosurgery and laser treatment might be performed if bleeding or cosmetic concerns exist. Since the patient in this case report was suffering from bleeding and itching, excision of the lesion was necessary for healthy sexual activity. As a result, the whole labia major and vulva on the right side were excised. A V-Y gluteal fold advancement flap was preferred for the reconstruction of this area. Bleeding of this type of lesion after the operation requires caution. This flap usually does not result in complications during surgery due to its thin and safe characteristics and its skin quality (6). Although Fordyce angiokeratoma has been reported in adult patients, this is the first childhood case reported in the literature in which reconstruction of the vulva was performed (7). Therefore, we believe that our case and its treatmentmerits special attention and should be reported due to its rarity.
